# Effective altruism and the dark side of entrepreneurship

**DOI:** 10.3389/fpsyg.2023.1247331

**Published:** 2023-10-16

**Authors:** Michael Olumekor, Muhammad Mohiuddin, Zhan Su

**Affiliations:** ^1^Graduate School of Economics and Management, Ural Federal University, Yekaterinburg, Sverdlovsk Oblast, Russia; ^2^Department of Management, Faculty of Business Administration, Laval University, Quebec, QC, Canada

**Keywords:** effective altruism, dark triad behavior, dark personality, entrepreneurship, Machiavellianism, narcissism, psychopathy

## Abstract

**Purpose:**

Effective Altruism (EA) has become one of the most prominent socio-philosophical movements of recent years. EA is also facing intense scrutiny due to the business practices of some of its most prominent adherents. On the other hand, the dark triad traits of Machiavellianism, narcissism and psychopathy have been getting increasing attention in entrepreneurship research. There is growing evidence that these traits can motivate entrepreneurial intention. We therefore sought to investigate if there was a connection between the entrepreneurship discourse in EA and traits corresponding to dark triad behavior.

**Design/methodology/approach:**

Using a discursive analytic method, we investigated the discursive threads on entrepreneurship in EA over a 10-year period.

**Findings:**

While we believe EA brings a much-needed perspective to the overall debate on doing good, we found ample evidence that it might have promoted the sort of dark triad behavior which some evidence suggests can lead to financial success, but can equally lead to the type of morally bankrupt, unethical and even illegal practices of some entrepreneurs. We also discovered a somewhat temporal dimension in EA’s discourse on entrepreneurship, beginning with discourse encouraging some risk taking and entrepreneurship, before moving on to discourses on the benefits of having a smart and illicit character, and ending with a focus on aggressive risk taking.

**Originality:**

The findings contribute to the still nascent debate on dark personality traits in entrepreneurship, and enriches the theoretical advancement of the field. However, our research differs from prior studies which were almost exclusively focused on the firm. Instead, we examine this phenomenon within a highly influential belief system/philosophical movement.

## 1. Introduction

There is considerable evidence of the benefits of entrepreneurship for economic growth and development (Audretsch et al., 2006). However, in recent decades, Silicon Valley style technology entrepreneurship – also referred to as tech entrepreneurship or techno-entrepreneurship in this paper – has become one of the most influential models of entrepreneurship (Suzuki et al., 2002). Countries, cities and governments around the world have sought to emulate or replicate it (OECD, 2010), such that hitherto successful entrepreneurial regions are disregarded because they are not involved in the “endless creation of highly innovative, technology-oriented, venture capital-backed gazelles and unicorns” (Pahnke and Welter, 2019). Moreover, this type of entrepreneurship has also become dominant in academic research where high growth and innovative enterprises are exulted (Audretsch, 2021). Other types of entrepreneurship such as necessity based, sole proprietor and self-employed entrepreneurs are often disparaged (Welter et al., 2017). In fact, some studies have gone further to argue that other types of small businesses and startups are less entrepreneurial if their founders do not achieve billionaire status (Henrekson and Sanandaji, 2014). Nevertheless, some scholars have begun to push back on this model of entrepreneurship, arguing for a more inclusive approach that appreciates the contributions of other types of entrepreneurship (Welter et al., 2017), and the rising social challenges which the Silicon Valley model has been unable to solve (Audretsch, 2021).

While there is little doubt in the financial and innovative benefits of tech entrepreneurship, there is an increasing body of evidence that this approach, which places persistent growth above anything else, is not just incapable of solving the socio-economic problems facing the world, but it actively creates them. For example, Facebook played a direct role in the devastating genocide of the Rohingya people (Yue, 2019), and there is mounting evidence of the negative psychological impact of social media usage on children and adolescents (Marino et al., 2018; Keles et al., 2020). In addition, the products created by these entrepreneurs are posing enormous danger to nature and the future of humanity (Mora et al., 2018; Creutzig et al., 2022), and threaten the very foundations of democracy (Tucker et al., 2017; Fukuyama et al., 2021). This paper does not seek to downplay the extraordinary achievements of Silicon Valley or tech entrepreneurs. It however argues that these types of entrepreneurs wield immense power and influence in present society, and would normally face ethical quandaries with a consequential impact on the rest of society. Therefore, as technology companies begin an arms race toward superiority in Artificial Intelligence (AI), understanding the motivations and character of the sort of people who become tech entrepreneurs is crucial. Some evidence show that the nature of the Silicon Valley model can encourage what a recent investigative analysis described as the “Machiavellian narcissists” (Griffith, 2023). High profile scandals have plagued some of the most celebrated tech entrepreneurs including the founders of WeWork, Uber, FTX, and numerous other lesser known companies (Griffith, 2023).

One of the earliest investigations of this phenomenon was the analysis of Maccoby (2000), who observed that leaders of the dot com boom were far more willing to court media attention that those of previous generations. This type of obsessive need for self-admiration and the limelight meets the Freudian assessment of a narcissistic personality (Freud, 1925; Maccoby, 2000; Rosenthal and Pittinsky, 2006). Narcissism, Machiavellianism and psychopathy have become known as the dark triad traits (Paulhus and Williams, 2002). Power/control – manipulation – and callousness are at the heart of all three traits (Furnham et al., 2013; Jones and Figueredo, 2013), and entrepreneurs can be motivated by a need to acquire power and domination over others (Brownell et al., 2021). This behavior is not exclusive to tech entrepreneurship. However, as technology continues to play an outsized role in the modern economy, and as the financial and power incentives of pursuing tech entrepreneurship continues to increase, tech entrepreneurship might be more appealing to these types of characters.

Effective Altruism (EA) has become one of the most influential belief systems of the twenty first century (Ackermann, 2022). Seeking to revolutionize charitable contributions and the debate around improving the world, EA has faced both praise and criticism in academic literature (Berkey, 2018; Broi, 2019; Dietz, 2019). EA is particularly influential in tech entrepreneurship with some of the most influential tech entrepreneurs in the world, including the founders of companies like Facebook, Instagram, Skype, PayPal, and Tesla among others, expressing some degree of devotion to the movement (Bennett et al., 2016; Ackermann, 2022). Moreover, EA consistently encourages its followers to pursue tech entrepreneurship and ranks it as its most important career choice.^1^ Since late 2022, EA has faced intense backlash due to the criminal investigation and subsequent criminal indictment of arguably its most prominent follower, Sam Bankman-Fried. Consequently, the goal of this research is to investigate the discursive threads in EA literature on Entrepreneurship, and specifically analyse it for dark triad behavior. This paper employs a discursive psychological approach grounded in the theoretical foundations of Potter and Wetherell (1987), Fairclough (1993), and Wiggins (2009), all of whom believed in the potent power of words to directly influence action and shape reality. The paper differs from prior studies by attempting to connect philosophical belief systems/practice to dark triad behavior in entrepreneurship, and therefore provides an important contribution to the contemporary debate on entrepreneurial behavior. The next section includes a theoretical background followed by the methodology, results, discussion, and conclusion/limitation.

## 2. Theoretical background

### 2.1. Effective altruism

According to MacAskill (2019), Effective altruism is a philosophical and social movement which promotes “the use of evidence and careful reasoning to work out how to maximize the good with a unit of resources.” Other scholars have expanded it to mean a framework for identifying the greatest potential benefits from any type of investment (Garrett et al., 2020). Since the movement took off in the late 2000s, it has set out to revolutionize the approach people take to charitable donations, career choice and overall living (Singer, 2010, 2015; MacAskill, 2015). While some scholars have linked EA to utilitarianism, others have contested this, arguing instead that EA is focussed on connecting moral decision making with scientific evidence (Kumar, 2020). In academics, EA has been used to analyse animal welfare, organ donation, clinical practice, and climate change, among others (Tonkens, 2018; Garrett et al., 2020; Freeling et al., 2022). EA also provides career advice to its adherents, asking them to choose careers that can bring about the most good in the world. However, careers such as working for non-profit organizations are rarely encouraged. Instead, proponents of EA argue that taking up careers in places such as banking and hedge funds can be more impactful, if people donate a portion of their wages to effective charitable causes (MacAskill, 2015). Entrepreneurship, tech entrepreneurship in particular, is a highly recommended career choice due to the potential financial gain it can generate.

Nevertheless, EA has faced vociferous criticisms since its inception. The most dominant criticism is its relegation of institutional change and socio-economic action, all of which critics argue have the most profound impact on improving the world (Herzog, 2015; Srinivasan, 2015; Dietz, 2019; Syme, 2019). Others have raised concern with the central premise of EA, which presupposes that the magnitude of impact of charitable organizations will always remain the same, and that tiny, incremental donations will make little difference in the organization’s impact. Instead, they argue that some organizations, such as those working against oppressive cultural norms, create “lumpy benefits on the path to fundamental change” (Côté and Steuwer, 2023). EA has also been accused of elitism, and in the wake of the collapse of FTX, the cryptocurrency firm founded by one of EAs most prominent members, it has faced intense scrutiny in popular media (Bush, 2022; Táíwò and Stein, 2022).

### 2.2. Dark triad behavior in entrepreneurship

Studies on entrepreneurship have demonstrated the enormous positive impact it can have on society. For example, entrepreneurship can promote job creation and economic growth (Cumming et al., 2014; Fritsch and Wyrwich, 2017), improve the public sector (Hayter et al., 2018; Olumekor, 2022), foster regional development (Fritsch and Mueller, 2004; Olumekor et al., 2023), and help to reduce poverty (Bruton et al., 2013). However, entrepreneurs have also engaged in morally bankrupt, unethical and outright illegal behavior (Machan, 1999; Fisscher et al., 2005; Baucus and Mitteness, 2016). The Enron fiasco, and the criminal prosecution of Purdue Pharma – and the Sackler family – for the opioid epidemic are some examples of this (Department of Justice, 2020). All these have prompted persistent investigations into the type of people who become entrepreneurs (Baum et al., 2007). While studies have long alluded to the positive motivations for entrepreneurship such as freedom/autonomy (van Gelderen and Jansen, 2006), there is now an increasing body of research connecting entrepreneurial intention to darker intentions. The dark triad traits in particular have become very prominent in recent decades. According to Paulhus and Williams (2002), the dark triad comprises the three distinct yet overlapping traits of Machiavellianism, narcissism, and psychopathy. A recent review revealed that people with dark triad traits are often attracted to entrepreneurship (Brownell et al., 2021). And other studies have confirmed that individuals high in one or more of the dark traits often choose to engage in entrepreneurship (Wales et al., 2013; Wu et al., 2019; McLarty et al., 2021). While manipulation, exploitation and domination are common across all three dark triad traits, studies have shown them to be different from each other (Paulhus and Williams, 2002).

#### 2.2.1. Machiavellianism

According to Dahling et al. (2009), Machiavellianism is a character which reflects a desire to manipulate and control others to pursue personal goals. They are self-centred, demonstrated by their need to seek personal status, and possess a propensity to distrust others. Other studies have shown people showing high levels of Machiavellianism to be manifestly amoral, emotionally detached, good readers of social situations, and willing to be aggressive in the pursuit of personal goals (Christie and Geis, 1970; Czibor and Bereczkei, 2012; Al Aïn et al., 2013). These skills can make them particularly suited to entrepreneurship and business. Entrepreneurship can provide a platform for them to pursue personal status in the form of money and power (Zettler and Solga, 2013; Wu et al., 2019), and their manipulative skills, love of competition, aggressive risk-taking and adaptability can not only attract them to entrepreneurship but make them succeed in an overall business environment (Czibor and Bereczkei, 2012; Brownell et al., 2021). In addition, their willingness to do anything it takes to succeed including breaking the law and taking unethical approaches can provide short term success in business settings (Al Aïn et al., 2013; Hmieleski and Lerner, 2016; Wu et al., 2019).

#### 2.2.2. Psychopathy

Psychopathy is characterized by problems with empathy and emotional intelligence (Hare, 2003; Mullins-Sweatt et al., 2010). This makes people with high levels of psychopathy think little before taking advantage of people to further their own agenda. Having low emotional intelligence also means that psychopaths experience less fear in situations that would normally be highly stressful for others (Paulhus and Williams, 2002; Brownell et al., 2021). Therefore, psychopaths enjoy risk taking activities and pursue excitement and stimulation (Wu et al., 2019). People exhibiting high levels of psychopathy can be charming people but are also ruthless, extremely arrogant and drawn to positions of power (Boddy, 2015). They experience little guilt and rarely take responsibility for their actions (Brownell et al., 2021). They are also willing to disregard rules and norms as they consider themselves superior to them (Wu et al., 2019). Studies have shown all of the aforementioned traits of psychopaths, coupled with a high degree of conscientiousness, can make them thrive in entrepreneurship or business contexts (Mullins-Sweatt et al., 2010; Kraus et al., 2018). Entrepreneurship can be particularly appealing to psychopaths because it eliminates the burden of following the orders of others, and grants them a great degree of power and control. Furthermore, the ruthlessness, risk-taking and cunning abilities of psychopaths can make them thrive in an entrepreneurial environment.

#### 2.2.3. Narcissism

Narcissism reflects a character with an exaggerated but fragile notion of self-importance and influence (Paulhus and Williams, 2002; Wales et al., 2013). They possess grandiose belief systems and ideas, and often lack empathy (Rosenthal and Pittinsky, 2006). It differs from the other dark triad traits of Machiavellianism and psychopathy mainly due to the narcissist’s feelings of superiority and entitlement (Wu et al., 2019). Furthermore, of the three traits, individuals with high levels of narcissism are more likely to maintain a good social relationship, at least for a short period of time (Jonason and Schmitt, 2012; Brownell et al., 2021; Gubik and Vörös, 2023). They are charismatic, extraverted, and seek attention and validation from others (Twenge et al., 2008). Studies have shown an increase in narcissism in recent years (Twenge et al., 2008). And according to Maccoby (2000), narcissist can be attracted to entrepreneurship due to the very high profile they enjoy in today’s society and the opportunity entrepreneurship provides to shape public opinion and leave a grand legacy of achievement. In addition, studies have shown that the need for power, achievement and superiority over others can motivate narcissists to be workaholics, which can in turn lead to success in entrepreneurship (Clark et al., 2010; Wales et al., 2013). However, other studies have shown that the need for attention in narcissism often leads to bold and aggressive actions which promotes extreme performance comprising of either big wins or big losses (Chatterjee and Hambrick, 2007).

## 3. Methodology

### 3.1. Research questions

This article adopts a discourse analytic approach (Potter and Wetherell, 1987) to investigate the entrepreneurship discourse of the EA movement. Our research sought to answer the following research questions:

RQ_1_: What are the main threads and content in EA discourse on entrepreneurship?

RQ_2_: Does the entrepreneurship discourse in the EA movement encourage dark triad behaviour?

Discourse analysis is a widely used theoretical and methodological approach in social psychology which argues that all forms of talk are important in shaping human life and decisions (Potter and Wetherell, 1987; Fairclough, 1993; Jørgensen and Phillips, 2002; Potter, 2003). According to Potter and Wetherell (1987), discourses do not just explain things, the do things and produce direct material effects on people. Furthermore, discourse is social action which “does not merely reflect reality, rather, it constructs reality in particular ways” (Wiggins, 2009). Therefore, discourse analysis examines how words are used to perform and influence action, and how language can subtly alter perceptions and make things happen (Brown and Yule, 1983; Potter and Wetherell, 1987). In the organizational and entrepreneurial context, studies are “increasingly conceptualizing societies, institutions, and identities as discursively constructed” (Hardy, 2001). It has been used to conceptualize entrepreneurship (Berglund and Johansson, 2007), examine gender discrimination (Ahl H. J., 2002; Ahl H., 2004), explain power relations (Mumby and Stohl, 1991), and investigate strategic management (Phillips et al., 2008), among others.

Our data collection and analysis were influenced by four main principles. First, that language and words are among the “most important phenomenon, accessible for empirical investigation, in social and organizational research” (Alvesson and Karreman, 2000). Second, that words are active and influence human action (Potter and Wetherell, 1987; Potter, 2003). Third, that discourse is a process of social interaction. Thus, the meaning of words can be found in the “disjuncture between dominant readings and individual interpretations” (Mumby, 1997), or as Wiggins (2009) described, what we mean by a statement is “not a matter of what we thought about when we said it, but how the words are interpreted and responded to by others.” Fourth, that the psychological traits of entrepreneurs can be different from others, and they can be influenced by darker motivations (Rauch and Frese, 2007; Frese and Gielnik, 2014; Brownell et al., 2021).

### 3.2. Data collection and analysis

The goal of this paper is to investigate the discursive content on Entrepreneurship within the EA community. To achieve this, a search for data was conducted on the 80,000 h website (80000hours.org) on the 16th of January, 2023. 80,000 h was created by two of the founders of the EA movement and is affiliated with the Oxford Uehiro Centre for Practical Ethics within the Faculty of Philosophy at Oxford University. The organization’s website serves as the unofficial content page of the EA movement, providing research/content on pressing issues such as career advice for early career members, and it wields considerable influence within the EA community. The discursive content includes blog posts, articles, podcasts/interviews, and career review, among others, making it a rich source of data collection. To collect data from the 80,000 h website, we painstakingly pored through hundreds of hours of podcasts content, dozens of pages of various content including blog posts/articles and career review. Due to the wide range of themes on the website, we were immediately focussed on extracting discourses solely focussed on entrepreneurship. Consequently, we searched for similar/alternate iterations of the concept including *entrepreneur*, *found*, *founder*, *start-up*, *ambition*, and *risk taking*. We then thoroughly read or listened to the content, and excluded those on non-profit organizations or social enterprise, while narrowing down to those on traditional profit-making entrepreneurship. In addition, we excluded discourses exclusively dedicated to the biography or salary of entrepreneurs. Therefore, contents such as *How much do Y Combinator founders earn?* Or *Biographies of Top Entrepreneurs were excluded*. To be included in the results, publications must be directly focussed on the idea of profit making entrepreneurship or starting a business (Schumpeter, 2003).

An initial pool of 44 documents were retrieved from the 80,000 websites including audio and text data (*n* = 44). Following this, all podcasts/audio were transcribed to text and all 44 documents were processed using Microsoft Word. Then, we carried out a corpus analysis using the Tidytext text mining package in the statistical programming tool, R. The result of this analysis was triangulated with a manual analysis of the text. Thirty-one documents were removed for not discussing entrepreneurship in any relevant detail. In total, 13 documents (*n* = 13), covering dozens of pages, were included in the final data set for analysis. We re-read the texts of all 13 documents, cross analyzed them, and used a data extraction form to retrieve the results (Supplementary Table S1).

## 4. Results

We clustered our findings by mapping the discourse timeline before dividing them into the three main phases presented below.

### 4.1. Phase 1: Encouraging risk taking and entrepreneurship (2012–2013)

The first significant content on entrepreneurship was the 2012 blog article titled: *Salary or startup? How do-gooders can gain more from risky careers*. The article focussed on the dilemma between working for a company and starting a business. It particularly highlighted the reasons taking big risks with entrepreneurship might be the biggest way to make an impact on the lives of others:

“But given the sheer size of the wedge between raw expected returns and risk-adjusted returns, it will sometimes be enough to make risk-taking and entrepreneurship the winning choice”.^2^

“Thus, even though most firms failed, a randomly selected team of startup founders (with some VC funding) would on average earn $9.2 million by exit (IPO, sale of the company, or failure).” (see footnote 2).

The main rationale for this is that even failing on a startup can potentially earn the entrepreneur a lot of money. This sentiment was expressed consistently across various texts and documents. Entrepreneurship was highlighted as an excellent choice for people planning to earn a lot of money. The potential to donate these earnings to charitable causes was promoted as a reason for this:

“If your aim is to maximise your earnings to maximise your charitable donations, you might want to consider entrepreneurship”.^3^

In this first phase, the dominant discourse was on encouraging entrepreneurship among the EA community, and justifying the benefits of pursuing entrepreneurship as a career choice. It also includes discourses on the benefits of risk taking, grit and having a technical background for succeeding in entrepreneurship.

### 4.2. Phase 2: Break the rules, ‘smart and illicit’ (2014–2016)

From 2014, we found a significant shift in the discursive rhetoric of 80,000 h. Prior to 2014, discourses highlighted the benefits of taking risks with entrepreneurship and the potential to earn a lot of money. Subsequent discourses from 2014 began to promote a darker type of entrepreneurial character, justifying them with research studies such as those by Wasserman (2006) and Levine and Rubinstein (2013, 2017).

For example, in *What I learned quitting my job to found a tech start up*, gaining power and control were discussed as the primary motivation for becoming an entrepreneur:

“The founder’s dilemma contrasts the two basic motivations of entrepreneurs: to make a lot of money and to be in a position of power. Noam Wasserman calls these outcomes “Rich” and “King”. The “Rich” goals have been elaborated on before, but I would like to give a brief summary of some reasons why King outcomes are interesting for EAs”.^4^

“My direct employment career path was a progression from “writing code” to “telling people to write code”.… It turns out that I actually enjoy some of these things and would probably pursue them even if I decide startups aren’t a good idea.” (see footnote 4).

The discourse also began to call more attention to, and encourage a particular type of entrepreneurial character: the ‘smart and illicit’ type. According to Levine and Rubinstein (2013), this type of entrepreneur is a “person who tends to “break the rules” (as measured by the degree to which the person engaged in illicit activities before the age of 22) who is especially likely to become a successful entrepreneur.” References to this were made across several discourses:

“I’m hesitant to describe what it’s like running a company, because my description will be “hard” and then we will have even more of a bias towards overconfident people starting companies. Additionally, the things which seem to indicate that a person will be a good entrepreneur verge on the tautological… One interesting predictor though is a “break the rules” attitude.” (see footnote 4).

“Successful tech entrepreneurs are very intelligent, motivated, deeply interested in entrepreneurship, and willing to break the rules”.^5^

Discourses went further to encourage people with the ‘smart and illicit’ mindset to pursue entrepreneurship:

“In fact, the people who most exemplify these traits – what the authors call “smart and illicit” – tend to get the biggest earnings boost from switching to self-employment”.^6^

“We also saw earlier that those who have the combination of “smart and illicit” traits tended to do better when they made the switch, so especially consider it if you’re in that group.” (see footnote 6).

By the end of this phase, the smart and illicit trait had become one of the most dominant qualities of a successful entrepreneur within EA discourse. Crucially, none of the examined discourse included any reference to the consequences or possible harm that could arise from illicit behavior.

### 4.3. Phase 3: Taking aggressive risks (2017–2022)

The main entrepreneurship discourse between 2017 and 2022 was the podcast interview titled: *Sam Bankman-Fried on taking a high-risk approach to crypto and doing good*. In this phase, the discourse was focussed on taking aggressive risks and the benefits it can provide to the world:

“If your goal is to have impact on the world — and in particular if your goal is to maximize the amount of impact that you have on the world — that has pretty strong implications for what you end up doing. Among other things, if you really are trying to maximize your impact, then at what point do you start hitting decreasing marginal returns? Well, in terms of doing good, there’s no such thing: more good is more good”.^7^

“That means that you should be pretty aggressive with what you’re doing, and really trying to hit home runs rather than just have some impact — because the upside is just absolutely enormous.” (see footnote 7).

The interviewer went even further by encouraging people to be more open to taking what he termed “radical gambles”:

“So you kind of want to just be risk neutral. As an individual, to make a bet where it’s like, “I’m going to gamble my $10 billion and either get $20 billion or $0, with equal probability” would be madness. But from an altruistic point of view, it’s not so crazy. Maybe that’s an even bet, but you should be much more open to making radical gambles like that.” (see footnote 7).

In summary, our results show that discourse on entrepreneurship began as a way to encourage EA believers to earn a lot of money which can then be donated toward charitable causes. As such, the first phase included discourse on the benefits of taking risks and choosing tech entrepreneurship. However, discourses in subsequent phases began to shift toward promoting traits of entrepreneurship specific to the dark triad character. This culminated in the interview with Sam Bankman-Fried where the Machiavellian *end justifies the means* approach was dominant.

## 5. Discussion

This research contributes to the existing studies on the dark triad behavior in entrepreneurship (Wales et al., 2013; Do and Dadvari, 2017; Brownell et al., 2021). Using a discursive psychology approach (Potter and Wetherell, 1987; Fairclough, 1993), we examined the main entrepreneurship discourses in EA literature to explore if it encouraged traits associated with the dark triad. To the best of our knowledge, our research represents the first attempt to investigate the dark triad behavior, not within the firm, but in a social/philosophical movement. EA has become one of the world’s most influential philosophical movements of the last decade, especially prominent among tech entrepreneurs. Our findings indicate that it was a mutual relationship as EA discourse intensely promoted tech entrepreneurship, ranking it as the best career choice for making the most difference in the world. Following the works of Fairclough who argued that discourse can be “simultaneously a piece of text, an instance of discursive practice, and an instance of social practice,” we thoroughly scrutinized the discourse on tech entrepreneurship and made a number of important discoveries.

Our analysis portrayed a temporal change in the discourse on entrepreneurship, beginning with discourses encouraging risk taking and entrepreneurship between 2012 and 2013, before moving on to discourse on the benefits of having a smart and illicit character, and ending with a focus on aggressive risk taking. Our results reveal that EA discourse contained the sort of aggressiveness which studies suggests are might be typical to all three personalities of the dark triad, but more particular to Machiavellianism and Narcissism (Paulhus and Williams, 2002). According to Brownell et al. (2021), people who possess Machiavellian personalities take an end justifies the means approach to business and tend to show strong adaptability, competitiveness and aggressive risk taking. They are also motivated by power and control, and are willing to use any necessary means to achieve their goals (Al Aïn et al., 2013; Zettler and Solga, 2013). This was most evident in the interview: *Sam Bankman-Fried on taking a high-risk approach to crypto and doing good*, where donating money to good causes was used to justify an aggressive form of risk taking which would eventually lead to the fraudulent indictment and criminal prosecution of the interviewee. In addition, having a direct impact on the world was among the factors used to dismiss legitimate concerns on the impact of the interviewee’s business on issues like climate change. Furthermore, other discourses included texts on how achieving domination (Brownell et al., 2021) – being motivated by power and control – was the primary motivator for starting a business, a trait typical of dark triad personalities (Jones and Figueredo, 2013; Hoang et al., 2022). In fact, studies have consistently shown domination to be among the few overlapping traits of people high on narcissism, Machiavellianism, and psychopathy (Raskin et al., 1991; Paulhus and Williams, 2002; Brownell et al., 2021).

Additionally, our findings show that one of the most dominant and encouraged character in EA discourse is the smart and illicit type of entrepreneur. References to this type of personality was made across several discourses, particularly in discourses between 2014 and 2016. According to Levine and Rubinstein (2013) (referenced in EA discourse): “It is the high-ability (as measured by learning aptitude and success as a salaried worker) person who tends to “break the rules” (as measured by the degree to which the person engaged in illicit activities before the age of 22) who is especially likely to become a successful entrepreneur.” This type of behavior is consistent with prior literature on the dark triad behavior in entrepreneurship. For example, while the emotional difficulty of psychopaths has received a lot of attention (Wu et al., 2019), studies have also shown them to be very smart and willing to break rules and norms to achieve success (Mathieu et al., 2013, 2014; Boddy, 2015). Moreover, research has shown that people with personalities like these tend to pursue entrepreneurship (Hmieleski and Lerner, 2016; Do and Dadvari, 2017; McLarty et al., 2021).

Although dark triad traits have been considered socially malevolent (Paulhus and Williams, 2002; Jones and Figueredo, 2013), there is some evidence linking them to entrepreneurial success (Wales et al., 2013; Gubik and Vörös, 2023), a fact which can explain why it was desirable in EA entrepreneurship discourse. However, there are significant challenges with encouraging dark triad behavior. First, as Boddy (2015) argued, dark triad behavior can potentially be linked to corporate fraud and the sort of behavior which led to the 2007 global financial crisis. Furthermore, while people with dark triad personalities can be found in successful ventures, they can also be found in prison settings (Mullins-Sweatt et al., 2010). The recent criminal indictment of arguably the most prominent proponent of EA is a prime example of this.

## 6. Conclusion and limitation

Our research investigates the ways in which entrepreneurship discourse in EA could have promoted traits characteristic to dark triad behavior. Using a discursive analytic approach, our findings reveal that domination, aggressiveness, and a tendency to break rules/norms were especially frequently highlighted in EA discourse as a prerequisite for entrepreneurial success. All three traits have been established as characteristic of dark triad personalities. Our study contributes to the growing debate on dark triad behavior in entrepreneurship. However, it differs from prior studies by exploring the topic not within a for-profit venture, but in a popular non-profit social/philosophical movement.

As the race toward artificial intelligence accelerates, tech entrepreneurs are bound to play an outsized role in shaping the future of humanity. Nevertheless, questions remain on the precise approaches to mitigate the dark triad behavior for the benefit of society. There is a clear need for introducing or strengthening existing ethical training for entrepreneurs. For example, universities can include stronger ethical curriculums to their entrepreneurship education programs, and incubators and accelerators can prioritize ethical training. Furthermore, while knowledge of narcissistic business leaders has existed for some time, research on the pervasiveness of dark triad traits in entrepreneurship is relatively nascent. Therefore, there needs to be an increase in awareness of the challenge and a provision of psychological support as part of the entrepreneurial process. This can be achieved with the support of investors and Venture Capital (VC) funds, who can emphasize psychological support as part of the fund-raising requirements for entrepreneurs. In addition, the level of ethical regulation, oversight and criminal prosecution has failed to keep up with the unethical behavior of entrepreneurs (Griffith, 2023). Policy-makers can consider strengthening this process, and improving the reporting standards for entrepreneurs.

Our research is limited in a number of ways. First, because the research topic was specific to entrepreneurship, we only included EA content exclusively focussed on entrepreneurship or the idea of starting a business venture. Second, the 80,000 h website was used as the primary source of data collection. Finally, the analysis and findings of this paper should not be taken as a clinical confirmation of the presence of dark triad behavior in the EA community.

## Data availability statement

All data used in this study can be found in the online repository of 80,000 hours: https://80000hours.org/topics/.

## Author contributions

MO was responsible for the conceptualization, data collection, data analysis, and writing the original draft of the manuscript. MM and ZS were responsible for revising the original draft. All authors read and approved the final version of the manuscript.

## Footnotes

^1^See for example Todd (2014), In which career can you make the most difference? *80,000 Hours*. Accessed April 15, 2023.

^2^https://80000hours.org/2012/01/salary-or-startup-how-do-gooders-can-gain-more-from-risky-careers/. Accessed January 16, 2023.

^3^https://80000hours.org/2013/09/should-more-altruists-consider-entrepreneurship/. Accessed January 16, 2023.

^4^https://80000hours.org/2014/10/what-i-learned-quitting-my-job-to-found-a-tech-startup/. Accessed January 16, 2023.

^5^https://80000hours.org/career-reviews/tech-entrepreneurship/. Accessed January 16, 2023.

^6^https://80000hours.org/2016/02/what-the-literature-says-about-the-earnings-of-entrepreneurs/. Accessed January 16, 2023.

^7^See for example Sam Bankman-Fried and Robert Wiblin (2022), Sam Bankman-Fried on taking a high-risk approach to crypto and doing good. Accessed January 16, 2023.
