# Supplementary material for: Effective altruism and the dark side of entrepreneurship
Source: Front Psychol. 2023 Oct 16;14:1247331. doi: 10.3389/fpsyg.2023.1247331 (PMC10614055; doi:10.3389/fpsyg.2023.1247331)
Supplement: Supplementary file 1 [file Table_1.docx]

Supplementary Material

Effective altruism and the dark side of entrepreneurship

Michael Olumekor, Muhammad Mohiuddin* and Zhan Su

*** Correspondence:** Muhammad Mohiuddin: muhammad.mohiuddin@fsa.ulaval.ca

# Supplementary Table 1: Entrepreneurship discourse in EA

| **#** | **Title** | **Year** | **Document type** | **Author(s)** | **Key texts (quotes)** | **Conclusion** | **URL links** |
| --- | --- | --- | --- | --- | --- | --- | --- |
| #1 | Salary or startup? How do-gooders can gain more from risky careers | 2012 | Blog post / article | Carl Shulman | “But given the sheer size of the wedge between raw expected returns and risk-adjusted returns, it will sometimes be enough to make risk-taking and entrepreneurship the winning choice.”  “Thus, even though most firms failed, a randomly selected team of startup founders (with some VC funding) would on average earn $9.2 million by exit (IPO, sale of the company, or failure).” | Take risks and win big | https://80000hours.org/2012/01/salary-or-startup-how-do-gooders-can-gain-more-from-risky-careers/ |
| #2 | Entrepreneurship: a game of poker, not roulette | 2012 | Blog post / article | Carl Shulman | “By paying attention to predictors of entrepreneurial success (whether good news or bad), you can better tell whether you have a winning hand or should walk away for a different game.” | Predictors of success | https://80000hours.org/2012/02/entrepreneurship-a-game-of-poker-not-roulette/ |
| #3 | Should more altruists consider entrepreneurship? | 2013 | Blog post / article | Jess Whittlestone | “if your aim is to maximise your earnings to maximise your charitable donations, you might want to consider entrepreneurship.” | Benefits of entrepreneurship. EAs should consider entrepreneurship | https://80000hours.org/2013/09/should-more-altruists-consider-entrepreneurship/ |
| #4 | Careers advice from top tech entrepreneur Marc Andreessen | 2013 | Blog post / article | Benjamin Todd (W/ insights from Marc Andreessen) | “Andreessen thinks that the best opportunities generally involve significant risk, so it’s difficult to have substantial achievements without taking on risk.  We think this is plausible. Some additional evidence is provided by high expected returns from start-ups, set against very high risks. Overall, however, we’re unsure about the extent to which taking on more risk will allow you to have more impact.” | Choose careers wisely | https://80000hours.org/2013/11/careers-advice-from-top-tech-entrepreneur-marc-andreessen/ |
| #5 | Interview with Matt Clifford | 2013 | Interview | Thomas Hendrey (W/ Matt Clifford (MC)) | “What we would really want to know is what they actually did. Failing because you couldn’t find a fit between the market you were trying and the product you were creating: that’s fine. That happens all the time. Failing because you haven’t really tried may be an indication of a more flaky personality which is a real problem.” (MC)  “I wouldn’t do an MBA unless you would do one anyway. In fact within the community it seems to be quite a negative signal. I think the reason is probably that MBAs teach you how to run large businesses and startups are not small versions of large businesses. They’re wildly different so most of what you learn is not that helpful.” (MC)  “To be successful you have to build something that solves something someone is already spending their time thinking is a problem. It’s hard to convince someone that something is a problem, they kind of already need to believe that they have one. So if you’ve been one of those people it is a whole lot easier.” (MC) | Becoming a tech entrepreneur | https://80000hours.org/2013/10/interview-with-matt-clifford/ |
| #6 | What I learned quitting my job to found a tech startup | 2014 | Blog post / article | Ben West | “The founder’s dilemma contrasts the two basic motivations of entrepreneurs: to make a lot of money and to be in a position of power. Noam Wasserman calls these outcomes “Rich” and “King”. The “Rich” goals have been elaborated on before, but I would like to give a brief summary of some reasons why King outcomes are interesting for EAs.”  “My direct employment career path was a progression from “writing code” to “telling people to write code”.… It turns out that I actually enjoy some of these things and would probably pursue them even if I decide startups aren’t a good idea. I never would’ve found that out at my previous job.”  “I’m hesitant to describe what it’s like running a company, because my description will be “hard” and then we will have even more of a bias towards overconfident people starting companies. Additionally, the things which seem to indicate that a person will be a good entrepreneur verge on the tautological… One interesting predictor though is a “break the rules” attitude”.  “People who both engaged in illicit activities as teenagers and scored highly on learning aptitude tests… tend receive much larger increases in earnings when they become incorporated self-employed business owners than people without that combination of traits.” (Levine and Rubinstein, 2017). | Be in control. Break the rules. | https://80000hours.org/2014/10/what-i-learned-quitting-my-job-to-found-a-tech-startup/ |
| #7 | Interview with Matt Gibb | 2014 | Blog post / article | Thomas Hendrey (W/ Matt Gibb) | “Definitely just start. Don’t do replacement activities. The quickest way to understand what you need to learn and what you need to do is by getting spanked by the market or getting spanked by the situation you need to solve and then just fixing it.” | Start a business. Learn by doing. | https://80000hours.org/2014/02/interview-with-matt-gibb/ |
| #8 | In which career can you make the most difference? | 2014 | Blog post / article | Benjamin Todd | “Tech entrepreneurship seems to be one of the highest-earning careers, offering great potential for earning to give”  “Entrepreneurship outside of technology also seems of high potential, but ranks lower in terms of expected earnings, usefulness of the skills and your chances of improving society with your products.” | Tech entrepreneurship is ranked the best career choice | https://80000hours.org/2014/02/in-which-career-can-you-make-the-most-difference/ |
| #9 | Found a tech startup | 08-2014 | Career review | Benjamin Todd | “Successful tech entrepreneurs are very intelligent, motivated, deeply interested in entrepreneurship, and willing to break the rules”  “It is a particular combination of traits that seems to matter for both becoming an entrepreneur and succeeding as an entrepreneur. It is the high-ability (as measured by learning aptitude and success as a salaried worker) person who tends to “break the rules” (as measured by the degree to which the person engaged in illicit activities before the age of 22) who is especially likely to become a successful entrepreneur.” (Levine and Rubinstein, 2013) | Benefits and traits of entrepreneurship | https://80000hours.org/career-reviews/tech-entrepreneurship/ |
| #10 | Podcast with Ben West, who expects to donate tens of millions for charity through tech entrepreneurship | 2015 | Podcast | Benjamin Todd (BT) (W/ Ben West BW)) | “I am very sceptical of the capital that actually starting a company provides, and I would definitely agree that working for a big company, or even a small one could be a much more valuable experience than starting your own” (BW).  *BT’s response:*  “The thing it does seems to have in its advantages is potentially learning a lot, in particular a rounder type of skill set” (BT) | Entrepreneurial life | https://80000hours.org/2015/12/interview-with-ben-who-expects-to-donate-eight-figures-for-charity-through-tech-entrepreneurship/ |
| #11 | What the literature says about the earnings of entrepreneurs | 2016 | Blog post / article | Benjamin Todd | “In fact, the people who most exemplify these traits – what the authors call “smart and illicit” – tend to get the biggest earnings boost from switching to self-employment”.  “We also saw earlier that those who have the combination of “smart and illicit” traits tended to do better when they made the switch, so especially consider it if you’re in that group.” | Entrepreneurs (especially illicit ones) earn more. | https://80000hours.org/2016/02/what-the-literature-says-about-the-earnings-of-entrepreneurs/ |
| #12 | Doing good through for-profits: Wave and financial tech | 2016 | Blog post / article | Robert Wiblin | “The most sticky moment was the first week of researching the idea when we talked to some Kenyans and described our idea, and they were very excited and said “this will grow like wildfire” and “when can I use it?” That was by far the most excited anyone had been about one of our startup ideas, and it was multiple people. We looked at each other and said “we have to make this work”.” | For profit entrepreneurship can be impactful | https://80000hours.org/2016/02/doing-good-through-for-profits-lincoln-quirk-and-wave/ |
| #13 | Sam Bankman-Fried on taking a high-risk approach to crypto and doing good | 2022 | Podcast | Robert Wiblin (RW), Keiran Harris (W/ Sam Bankman-Fried (SBF)) | “That means that you should be pretty aggressive with what you’re doing, and really trying to hit home runs rather than just have some impact — because the upside is just absolutely enormous.” (SBF)  “So you kind of want to just be risk neutral. As an individual, to make a bet where it’s like, “I’m going to gamble my $10 billion and either get $20 billion or $0, with equal probability” would be madness. But from an altruistic point of view, it’s not so crazy. Maybe that’s an even bet, but you should be much more open to making radical gambles like that.” (RW) | Take aggressive risks. There can only be upsides | https://80000hours.org/podcast/episodes/sam-bankman-fried-high-risk-approach-to-crypto-and-doing-good/ |

Source: Authors’ research. W/ = with. EA(s) = Effective Altruist(s).
